# Crosstalk between JNK and SUMO Signaling Pathways: deSUMOylation Is Protective against H_2_O_2_-Induced Cell Injury

**DOI:** 10.1371/journal.pone.0028185

**Published:** 2011-12-02

**Authors:** Marco Feligioni, Elisa Brambilla, Agata Camassa, Alessandra Sclip, Andrea Arnaboldi, Federica Morelli, Xanthi Antoniou, Tiziana Borsello

**Affiliations:** Department of Neuroscience, Mario Negri Institute for Pharmacological Research, Milano, Italy; Thomas Jefferson University, United States of America

## Abstract

**Background:**

Oxidative stress is a key feature in the pathogenesis of several neurological disorders. Following oxidative stress stimuli a wide range of pathways are activated and contribute to cellular death. The mechanism that couples c-Jun N-terminal kinase (JNK) signaling, a key pathway in stress conditions, to the small ubiquitin-related modifier (SUMO), an emerging protein in the field, is largely unknown.

**Methodology/Principal Findings:**

With this study we investigated if SUMOylation participates in the regulation of JNK activation as well as cellular death in a model of H_2_O_2_ induced-oxidative stress. Our data show that H_2_O_2_ modulates JNK activation and induces cellular death in neuroblastoma SH-SY5Y cells. Inhibition of JNK's action with the D-JNKI1 peptide rescued cells from death. Following H_2_O_2_, SUMO-1 over-expression increased phosphorylation of JNK and exacerbated cell death, although only in conditions of mild oxidative stress. Furthermore inhibition of SUMOylation, following transfection with SENP1, interfered with JNK activation and rescued cells from H_2_O_2_ induced death. Importantly, in our model, direct interaction between these proteins can occur.

**Conclusions/Significance:**

Taken together our results show that SUMOylation may significantly contribute to modulation of JNK activation and contribute to cell death in oxidative stress conditions.

## Introduction

Oxidative stress is involved in many diseases, such as Sickle Cell Disease, [1] atherosclerosis [2], Parkinson disease [3], myocardial infarction [4], Alzheimer disease [5], Ischemia [6], [7] Diabetes [8], Schizophrenia [9], Fragile-X syndrome [10] and Aging [11].

Oxidative stress is mediated by excessive exposure of cells to reactive oxygen species, which generate an oxidative burst of intracellular signaling cascades that induce cell death.

Among others, H_2_O_2_ induced oxidative stress leads to activation of c-Jun N-terminal kinase (JNK), a kinase that is strongly associated with many different stress stimuli and cell death [12], [13], [14], [15] as well as of the SUMOylation pathway, recently associated with ischemic events and cytoprotection [16], [17], [18], [19]. SUMO is a family of three proteins (SUMO-1, -2, and -3) that are involved in SUMOylation process, a posttranslational modification consisting of covalent conjugation of SUMO to target proteins. The SUMOylation cascade is ATP dependent. SUMO conjugates proteins through an enzymatic cascade similar to ubiquitination. The process involves a single SUMO-activating enzyme E1 (Uba2-Aos1), a SUMO-conjugating enzyme E2 (Ubc9) and often a SUMO E3 that facilitates the conjugation. A specific isopeptidase, member of the SENP family, ensures reversibility of the SUMOylation process [20], [21].

The role of SUMOylation in oxidative stress is yet to be defined. Nevertheless some very recent reports have shown a very intriguing link between JNK and SUMO signaling pathways in oxidative stress paradigm with H_2_O_2_ stimulation [22]. Other links between these two pathways have been reported. JNK activates the c-jun transcription factor while SUMOylation down-regulates it [23]. Additionally, SUMO inhibits the apoptosis signal-regulating kinase 1 (ASK1) activation, an upstream activator of JNK [24].

With this study we aim to elucidate the mechanism that couples JNK, a key kinase in cellular stress, to SUMO during H_2_O_2_-induced oxidative stress and clarify the impact of SUMOylation on cell death.

To explore the possibility that SUMOylation modulates JNK activity and consequently cellular death following oxidative stress, we stimulated human neuroblastoma SH-SY5Y cells with H_2_O_2_ and examined their activation pattern. To study the possible link between JNK and SUMO we over-expressed SUMO-1 or the de-SUMOylation enzyme catalytic sequence of SENP1 in SH-SY5Y cells.

We demonstrated a cross-talk between JNK and SUMO pathways. More specifically protein deSUMOylation prevented JNK activation and cell death. We provided also evidence for a potential interaction between P-JNK and SUMO-1.

## Results

### H_2_O_2_-induced activation of JNK in SH-SY5Y cells

In the first series of studies we examined the effect of increasing doses of H_2_O_2_ (10, 50, 75 and 100 µM) in cell death and JNK activation in undifferentiated human SH-SY5Y neuroblastoma cells.

**Figure 1:**
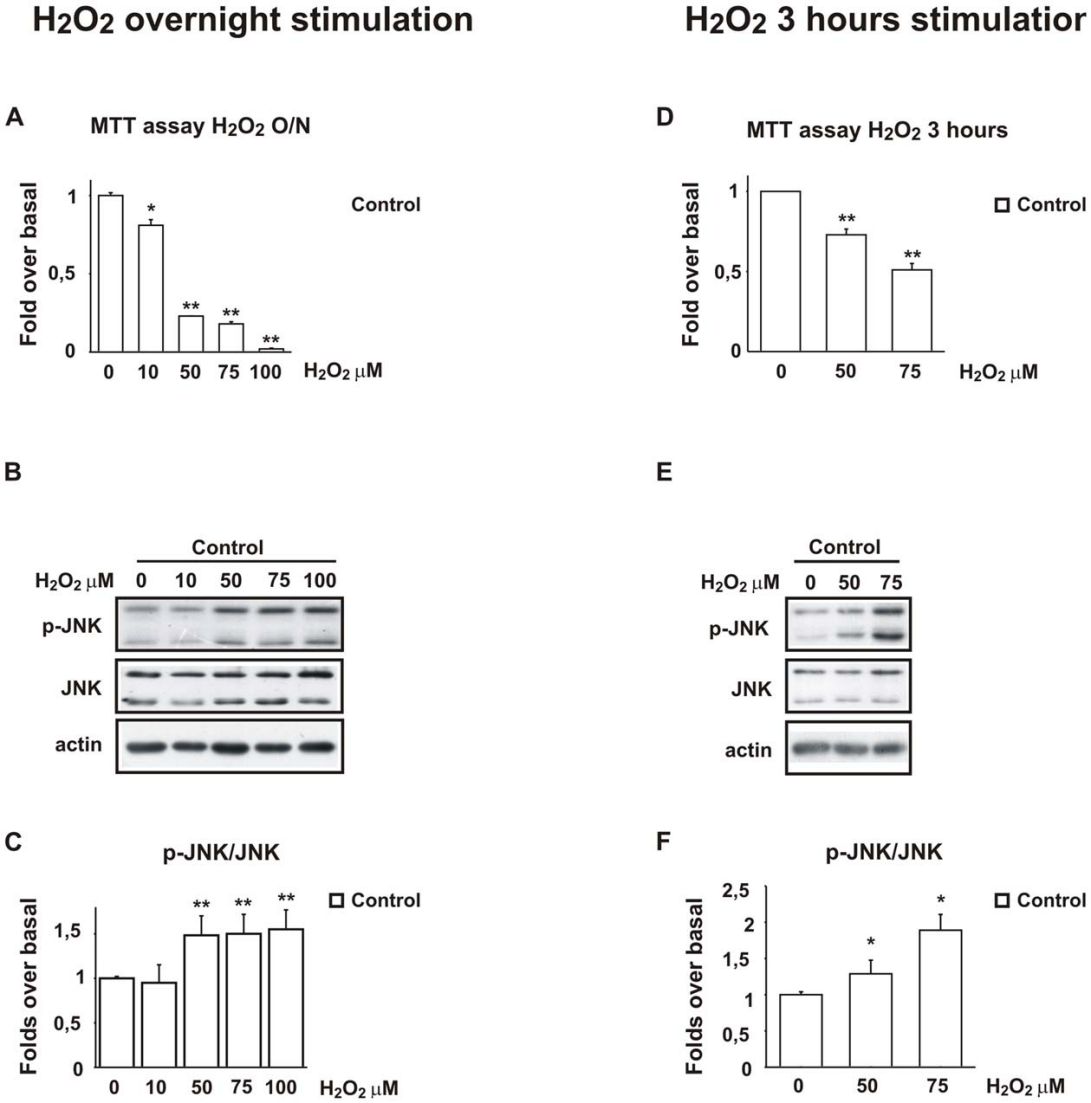
Cell death and JNK activation in SH-SY5Y cells stimulated with H_2_O_2_ overnight or for 3 h. **A**) Cell viability assay was performed employing MTT colorimetric method. A concentration dependent increase in cell death (80–100%) is observed following overnight H_2_O_2_ stimulation. Data are presented as fold increase. Values are from five experiments. *P<0.05, **P<0.01 vs control. **B**) Representative Western blot showing increase of P-JNK protein levels following overnight stimulation with increasing H_2_O_2_ concentrations qui. **C**) Densitometric analysis revealed a significant increase of P-JNK/JNK ratio following overnight H_2_O_2_ stimulation (50 mM H_2_O_2_: 1.31±0.12; 75 mM H_2_O_2_: 1.38±0.15; 100 mM H_2_O_2_: 1.40±0.16). Values are representative of five experiments and are expressed as mean±SEM. **P<0.01 versus control. **D**) Cell viability assay was performed employing MTT colorimetric method. A concentration dependent decrease in cell viability is observed following 3 h stimulation with H_2_O_2_ (50 mM: ∼30%; 75 mM: ∼50%). Data are presented as fold increase to controls and are representative of five experiments. **P<0.01 vs control. **E**) Representative Western blots showing increase of P-JNK protein levels following 3 h stimulation with increasing H_2_O_2_ concentrations. **F**) Densitometric analysis revealed a 1.28±0.18 and a 1.89±0.21 fold increase of P-JNK/JNK ratio following 3 h stimulation with 50 mM and 75 mM H_2_O_2_ respectively. Values are representative of four experiments and are expressed as mean±SEM. *P<0.05 vs control.

Cells exposed to H_2_O_2_ overnight (O/N) underwent abrupt shrinkage followed by cell death. As shown by the MTT cell viability assay, H_2_O_2_ (50 and 75 µM) induced approximately 80% cell death (50 µM H_2_O_2_: 0.23±0.01; 75 µM H_2_O_2_: 0.18±0.01) while 100 µM H_2_O_2_ induced 100% death (100 µM H_2_O_2_: 0.02±0.02) (Fig. 1A).

At the same time, H_2_O_2_ stimulation led to a marked increase in the phosphorylation status of JNK in a dose dependent manner (Fig. 1B, C).

In the second paradigm exposure of cells to 50 and 75 µM H_2_O_2_ for 3 h led to mild oxidative stress compared to overnight stimulation (Fig. 1D; 50 µM H_2_O_2_: 0.72±0.03; 75 µM H_2_O_2_: 0.51±0.04). Increase in JNK phosphorylation induced by H_2_O_2_ was concentration-dependent. 50 µM H_2_O_2_ led to a 1.28±0.18 fold of increase of the P-JNK/JNK ratio while a higher dose of 75 µM led to a 1.89±0.21 fold of increase in comparison to control non-stimulated cells (Fig. 1E, F).

### The cell permeable JNK inhibitor peptide D-JNKI1 prevents H_2_O_2_-induced cell death in SH-SY5Y cells

**Figure 2:**
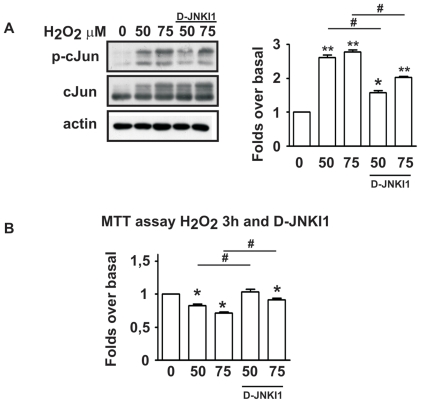
Transcription factor c-Jun activation and cell viability in SH-SY5Y cell line following stimulation with H_2_O_2_ for 3 h. **A**) Representative Western blot showing increase of P-c-Jun protein levels following H_2_O_2_ (50 and 75 mM) stimulation. Application of D-JNKI1, 30 min prior to H_2_O_2_ stimulation decreased P-c-Jun activation. Densitometric analysis revealed a fold increase ∼1.60 for 50 and ∼1.80 for 75 mM of P-c-Jun/c-Jun ratio in cells stimulated with H_2_O_2_ compared to control cells. D-JNKI1 inhibited activation of c-Jun by 0.57 fold for 50 and ∼1.00 for 75 mM. Data are from five experiments and values are expressed as mean±SEM. *P<0.05, **P<0.01 vs control and ^#^ *P*<0.05 vs H_2_O_2_ alone **B**) Cell viability assay was performed employing MTT colorimetric method. Application of D-JNKI1 rescued cells from H_2_O_2_ induced cell death. Data are presented as fold increase and are from five experiments. *P<0.05 vs control, ^#^ *P*<0.05 vs H_2_O_2_ alone.

To determine if JNK plays a pivotal role in H_2_O_2_-induced death cells were treated with D-JNKI1 (20 µM), a cell permeable JNK inhibitor [25], [26], 30 min before stimulation with H_2_O_2_. We then assessed the effect of D-JNKI1 on activation of the transcription factor c-Jun as well as on cell death. As shown in figure 2, stimulation with H_2_O_2_ activated c-Jun (P-c-Jun/c-Jun 50 µM H_2_O_2_: 2.61±0.07; 75 µM H_2_O_2_: 2.78±0.06). Instead, pre-treatment with D-JNKI1 prevented its activation (Fig. 2A, B- 50 µM H_2_O_2_: 1.57±0.05; 75 µM H_2_O_2_: 2.02±0.04). Most importantly, pre-treatment with D-JNKI1 completely prevented H_2_O_2_ induced death (Fig. 2B).

Altogether these results prove the pivotal role of JNK in H_2_O_2_ induced oxidative stress.

### Over-expression of SUMO-1+UBC_9_ and SENP1 plasmids in SH-SY5Y cell lines

**Figure 3:**
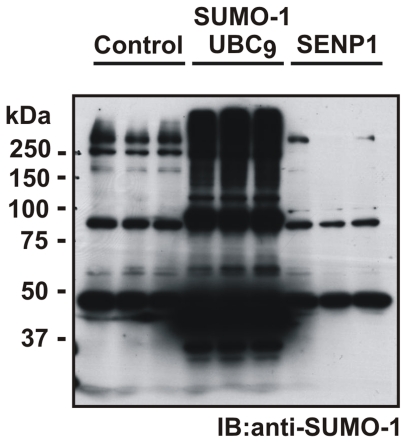
Successful transfection of SH-SY5Y cells with SUMO-1+UBC_9_ and SENP plasmids. Representative Western Blot showing transfection, in triplicate per condition, of SH-SY5Y cells with SUMO-1+UBC_9_ which leads to an increase in SUMOylation, while transfection of SH-SY5Y cells with SENP1 leads to complete abolishment of SUMOylation.

SH-SY5Y cell lines were successfully transfected with SUMO-1+UBC_9_ and SENP1 plasmids (YFP-SUMO-1, Flag- Ubc_9_ and GFP-SENP1). The expression level of SUMO-1+UBC_9_ was detected in cell lysates by Western blot using the anti-SUMO-1 antibody. A large number of SUMOylated proteins were detected compared to control SH-SY5Y indicating that the SUMO-1+UBC_9_ plasmid is functional. On the contrary a significant reduction in SUMOylated proteins is detected in cells in which SENP1 was over-expressed compared to untransfected cells indicating that the SENP1 plasmid is also functional (Fig. 3).

### SUMO-1+UBC_9_ over-expression increased H_2_O_2_-induced JNK activation in SH-SY5Y

It was recently shown that ROS-dependent JNK activation converges on the SUMO pathway [22] and we here tested the effect of SUMO-1+UBC_9_ over-expression on H_2_O_2_-induced activation of JNK.

SH-SY5Y cells over-expressing SUMO-1+UBC_9_ were stimulated with increasing concentrations of H_2_O_2_ (10, 50, 75 and 100 µM) O/N and 50, 75 µM for 3 h. Western blot analysis was employed to assess the role of SUMO-1 on JNK activation.

**Figure 4:**
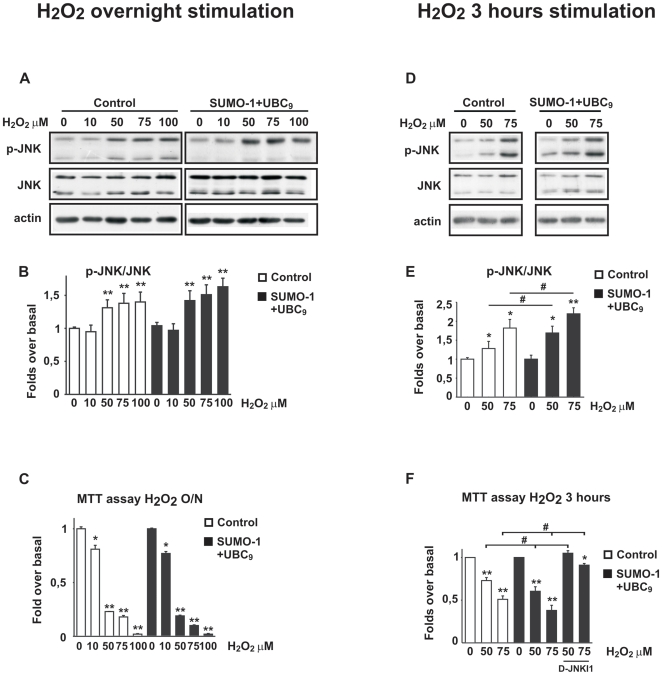
Effect of SUMO-1 over-expression on JNK activation and cell viability after overnight or 3 h stimulation with H_2_O_2_. **A**) Representative Western blot showing increase in P-JNK protein levels in cells overexpressing SUMO-1+UBC_9_ following overnight stimulation with H_2_O_2_. **B**) Densitometric analysis reveals that P-JNK/JNK ratio is not significantly increased in untransfected (white bars = Control) cells versus transfected (black bars = SUMO-1+UBC_9_) ones. Data are from four experiments and values are expressed as mean±SEM. **P<0.01 vs own control. **C**) Overnight H_2_O_2_ stimulation of transfected (black bars = SUMO-1+UBC_9_) cells did not lead to significant changes in cell death in comparison to untransfected (white bars = Control) ones, as assessed by MTT colorimetric assaying cell viability. Data are presented as fold increase and values are from five experiments. *P<0.05, **P<0.01 vs Control or H_2_O_2_ alone. **D**) Representative Western blot showing increase in P-JNK protein levels in cells overexpressing SUMO-1+UBC_9_ following stimulation with H_2_O_2_ for 3 h. **E**) Densitometric analysis reveals a significant increase of P-JNK/JNK ratio in transfected cells (black bars = SUMO-1+UBC9) compared to untransfected (white bars = Control) ones. Data are from five experiments and values are expressed as mean±SEM. *P<0.05 **P<0.01 vs Control or H_2_O_2_ alone and ^#^ *P*<0,05 Control versus SUMO-1+UBC_9_. **F**) Transfected cells (black bars = SUMO-1+UBC9) are more sensitive to H_2_O_2_-induced death compared to untransfected (white bars = Control) ones, as assessed by MTT colorimetric assaying cell viability. 20 mM of D-JNKI was applied 30 minutes before oxidative stress. D-JNKI1 rescued cells from H_2_O_2_- induced death. Data are presented as fold increase and values are from five experiments. **P<0.01 versus control, ^#^ *P*<0.05 H_2_O_2_ vs H_2_O_2_+D-JNKI1.

As shown in Fig. 1, P-JNK/JNK ratio increased following O/N stimulation with H_2_O_2_. SUMO-1+UBC_9_ over-expression did not affect JNK activation and did not prevent cell death (Fig. 4A–C).

On the contrary, SUMO-1+UBC_9_ over-expression led to a significant increase in JNK phosphorylation following stimulation of cells with H_2_O_2_ for 3 h. Specifically the P-JNK/JNK ratio increased by 1.69±0.17 and by 2.19±0.15 fold following stimulation with 50 µM and 75 µM H_2_O_2_ respectively (Fig. 4D, E).

Moreover, as shown in Fig. 4F, in the presence of SUMO-1+UBC_9_ cell viability was slightly worsen in both concentrations (50 µM H_2_O_2_: 0.60±0.05; 75 µM H_2_O_2_: 0.38±0.06) compared to control conditions. These data indicate that in conditions of mild oxidative stress SUMO-1+UBC_9_ over-expression contributes to JNK activation and cell death.

Interestingly, pre-treatment with D-JNKI1 powerfully reduced H_2_O_2_ induced cell death in SUMO-1 over-expressing cells (50 µM H_2_O_2_: 1.05±0.03; 75 µM H_2_O_2_: 0.91±0.02) (Fig. 4F), reinforcing the pivotal role of JNK in oxidative stress.

### SENP1 over-expression prevents H_2_O_2_-induced activation of JNK in SH-SY5Y

In light of the results obtained with SUMO-1+UBC_9_ over-expression we here tested the effect of de-SUMOylation on JNK activation and cell death.

**Figure 5:**
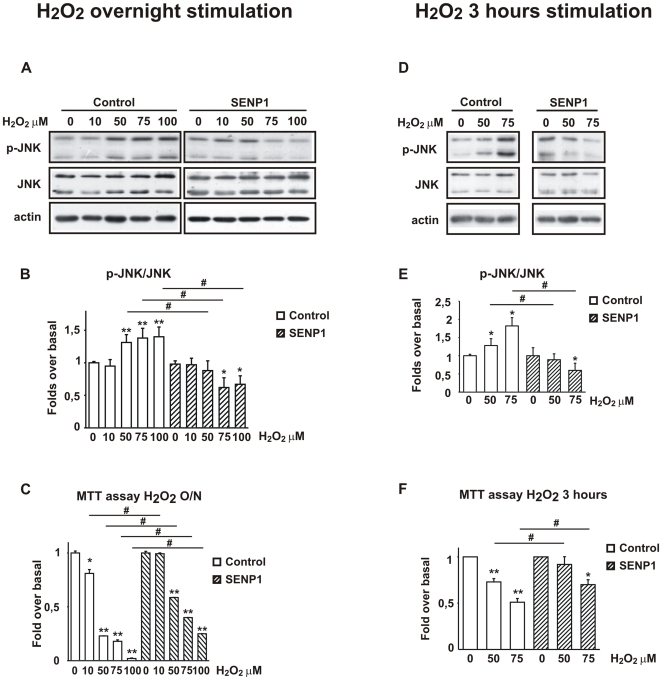
Effect of SENP1 over-expression on JNK activation and cell viability after overnight or 3 h stimulation with H_2_O_2_. **A**) Representative Western blot showing decrease in P-JNK protein levels in cells overexpressing SENP1 following overnight stimulation with H_2_O_2_. **B**) Densitometric analysis reveals that P-JNK/JNK ratio is significantly decreased in transfected (strapped lines bars = SENP1) cells compared to untransfected cells (white bars = Control). Data are from four experiments and values are expressed as mean±SEM. **C**) Transfected cells (strapped lines bars = SENP1) are more resistant to H_2_O_2_-induced death compared to untransfected (white bars = Control) ones, as assessed by MTT colorimetric assaying cell viability. Data are presented as fold increase and are from five experiments. **P<0.01 vs control ^#^ *P*<0.05 vs cells overexpressing SENP1. **D**) Representative Western blot showing decrease in P-JNK protein levels in cells overexpressing SENP1 and following stimulation with H_2_O_2_ for 3 h. **E**) Densitometric analysis reveals a significant decrease of P-JNK/JNK ratio in transfected cells (strapped lines bars = SENP1) compared to untransfected (white bars = Control). **F**) Transfected cells (strapped lines bars = SENP1) are more resistant to H_2_O_2_-induced death compared to untransfected (white bars = Control) ones, as assessed by MTT colorimetric assaying cell viability. Data are presented as fold increase and values are from five experiments. *P<0.05, **P<0.01 vs control, ^#^ *P*<0.05 control vs SENP1.

SH-SY5Y cells, over-expressing SENP1, were stimulated O/N with 10, 50, 75 and 100 µM H_2_O_2_. As shown in Figure 4 A, B, SENP1 over-expression led to a remarkable and significant dose dependent prevention of JNK activation compared to control (Fig. 5A, B). Notably, cells that over-express SENP1 were more resistant to oxidative stress compared to control (Fig. 5C).

SH-SY5Y cells over-expressing SENP1 were then stimulated with 50, 75 µM H_2_O_2_ for 3h (Fig. 5D, E). SENP1 SH-SY5Y stimulated with H_2_O_2_ presented a powerful reduction of P-JNK/JNK ratio (Fig. 5D). At 50 µM and of H_2_O_2_ and in the presence of SENP1 P-JNK levels were close to control levels (0.88±0.16) and were further reduced (0.59±0.19) with 75 µM of H_2_O_2_. Concomitantly, MTT-assay at 3 h denoted a strong protection from cell death in SENP1 over-expressing SH-SY5Y cells comparable to untransfected SH-SY5Y cells (50 µM H_2_O_2_: 0.91±0.08; 75 µM H_2_O_2_: 0.70±0.05) (Fig. 5F).

Altogether these findings indicate that SENP1 over-expression, and thus inhibition of SUMOylation prevents H_2_O_2_-induced cell death both O/N and at 3 h.

### P-JNK interacts with SUMO-1 in SH-SY5Y

To investigate whether P-JNK interacts with SUMO-1 we performed immunoprecipitation (IP) and immunostaining experiments.

**Figure 6:**
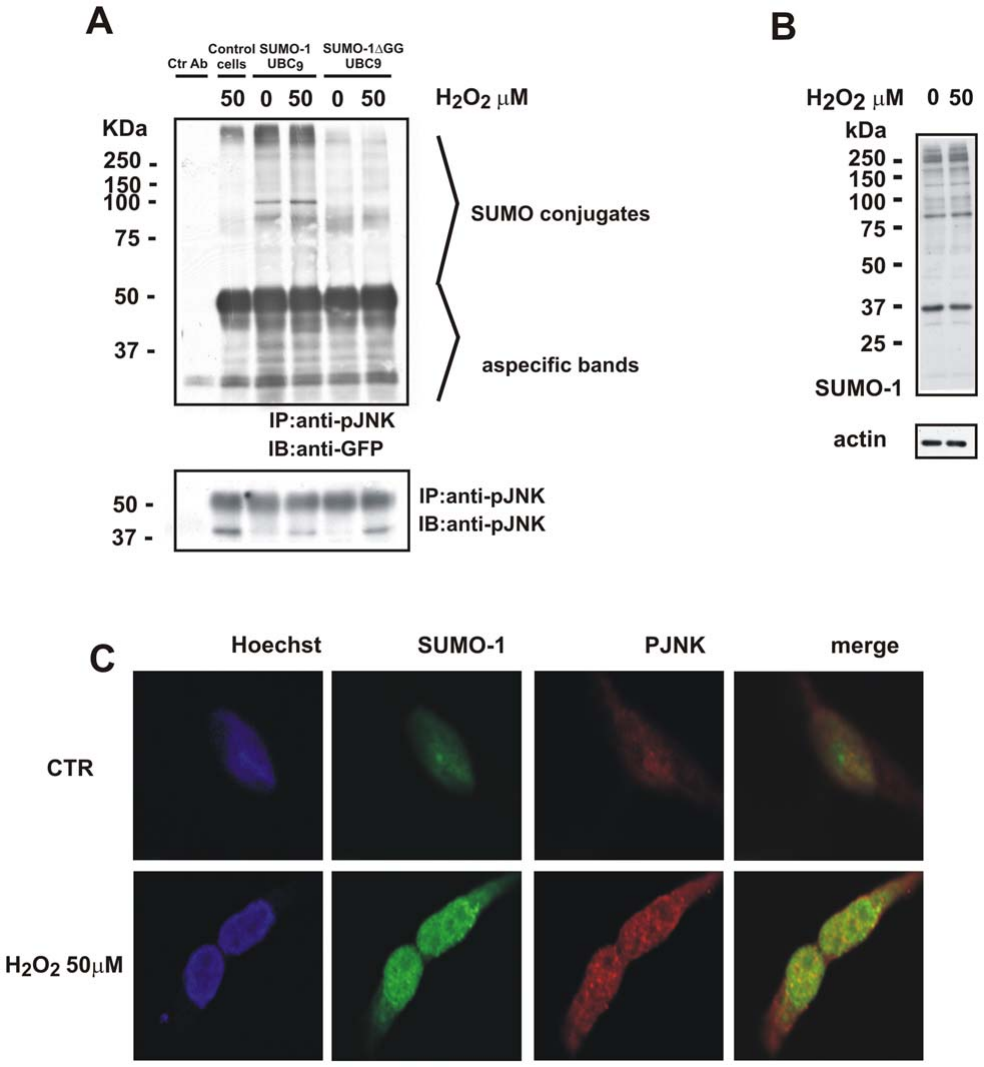
P-JNK and SUMO-1 interaction following stimulation with H_2_O_2_ for 3 h. **A**) Immunoprecipitation (IP) experiment. Untransfected (Control cells) or transfected (SUMO-1+UBC_9_ or SUMO-1DGG+UBC_9_) cells were stimulated with H_2_O_2_ (50 mM) and P-JNK was immunoprecipitated usind P-JNK antibody. Representative blot showing immunoprecipetated SUMOylated protein blotted with an anti-GFP antibody since SUMO constructs used were YFP tagged. Membranes were stripped and blotted with an anti-P-JNK antibody as control for the IP. Rabbit-IgG negative control antibody was used in the first lane (Ctr Ab). Bands below 50 kDa are IgG bands (50 and 25 kDa) and the others are unspecific signal of GFP antibody. **B**) Stimulation of untransfected SH-SY5Y cells with 50 or 75 mM of H_2_O_2_ did not affect the SUMOylated protein profile (from 250 to 37 kDa). **C**) Immunostaining shows that SUMO-1 (green) and p-JNK (red) colocalize in the nucleus following stimulation with H_2_O_2_ (50 mM) for 3 hours. Nuclei were stained with the nuclear marker Hoechst.

Cells were transfected with YFP tagged SUMO-1+UBC_9_ or YFP tagged SUMO-1DGG+UBC_9_ and stimulated with H_2_O_2_ (50 µM) for 3 hours. YFP tagged SUMO-1DGG plasmid encodes for a modified form of SUMO-1 in which the 2 glycine-residues responsible for protein SUMOylation bonds have been deleted resulting in a conjugation-deficient SUMO-1 protein. Cell extracts were immunoprecipitated with P-JNK antibody and immunoblotted with anti-GFP antibody, which recognises both SUMO-1 and SUMO-1DGG YFP tag. Association of P-JNK with SUMO-1 was detected both in control and in oxidative stress conditions. (Fig. 6A). SUMOylated proteins in SUMO-1 cells mildly increased by stimulation with 50 µM of H2O2. As expected, SUMOylation was drastically decreased in cells transfected with SUMO-1DGG both in unstimulated and stimulated conditions. Moreover, protein SUMOylation was absent in control conditions where cells were not transfected but still P-JNK is detectable. Such result denotes the specificity of the IP between P-JNK and GFP-tagged proteins. Aspecific protein immunoprecipitation by beads and antibody was verified using a control rabbit-IgG antibody for the IP (Fig. 6A, first lane Ctr Ab does not show any GFP immune reactivity).

Membranes were stripped and re-blotted for P-JNK to show the presence of active JNK in the lysates (Fig. 6A). As expected, in the presence of H_2_O_2_ phosphorylation of JNK was increased.

These data indicate that in SH-SY5Y cells over-expressing SUMO-1+UBC_9_, P-JNK interacts with SUMO-1. Such an interaction increases in stress conditions. Interestingly, as shown in Figure 6B, endogenous SUMOylation profile was not affected by H_2_O_2_.

Immunostaining further confirmed this result. In non transfected SH-SY5Y cells endogenous SUMO-1 (green) and P-JNK (red) are only mildly expressed (Fig. 6B, CTR). Instead H_2_O_2_ for 3 h enhanced the endogenous SUMO-1 signal and led to the formation of characteristic and previously described SUMO nuclear bodies (SNB) [27], [28]. Similarly, P-JNK is strongly induced by H_2_O_2_ and is principally localized in the nuclei of stimulated cells. In this condition, SUMO-1 and P-JNK colocalized (see merge) within the nucleus (Fig. 6B, 50 µM of H_2_O_2_).

## Discussion

JNK is a key enzyme in the cellular response to stress. Stress signals such as NMDA stimulation, Abeta fragments, hypoxia, reactive oxygen species, ultraviolet radiation, protein synthesis inhibitors can all activate JNK.

We found that H_2_O_2_-induced injury significantly increased JNK activation in SH-SY5Y. We proved that JNK plays a pivotal role in apoptosis, since the specific JNK inhibitor peptide, D-JNKI1, by inhibiting JNK action, totally prevents H_2_O_2_-induced death. These data are in agreement with our previous reports on the importance of JNK signaling in determining cell fate in stress conditions [14], [15], [29].

Several indications suggested that different stress-stimuli may link the JNK and SUMO pathways [23], [30], [31]. Similar to JNK, the SUMO pathway is also modulated by stress including heat shock, osmotic and oxidative stress (H_2_O_2_). Furthermore, SUMOylation is involved in the regulation of members of the JNK signaling cascade, including ASK, an upstream activator of JNK, and c-Jun, a major downstream target of JNK.

We here tried to determine whether JNK and SUMO-1 are interconnected in this model of oxidative stress.

To establish this crosstalk between JNK and SUMO-1 we over-expressed SUMO-1 in SH-SY5Y cells. Surprisingly over-expression of SUMO-1+UBC_9_ exacerbated the cellular-stress responses and co-participated with JNK in the apoptotic death mechanism induced by H_2_O_2_-stimulation. More specifically, SUMO-1+UBC_9_ over-expression increased JNK activation and H_2_O_2_ induced cell death at 3 h. Interestingly this mechanism seems to be more important in conditions of mild oxidative stress (3 h H_2_O_2_) since significant regulation was not observed following overnight stimulation. One could hypothesise that regulation of JNK activation by SUMO is an early event in the cell death pathway, which could explain why in extreme conditions, when cell viability is markedly decreased such a mechanism is no longer pivotal.

To better clarify the interaction between JNK and SUMO we over-expressed the catalytic portion of the deSUMOylating enzyme, SENP1, in SH-SY5Y cells and examined its role in H_2_O_2_ cell injury and JNK activation/regulation. We showed that SENP1 plays an intriguing role in this condition, since it is able to prevent JNK activation as well as cell death in both O/N and 3 h paradigms. This corroborates our previous finding and supports the idea that SUMO is not playing a protective role but instead participates with JNK in the stress cascade of H_2_O_2_-injury in SH-SY5Y. Consequently, by preventing SUMOylation, JNK activation is inhibited and prevents the specific cellular responses to mild as well as severe oxidative stress. To determine how P-JNK-SUMO interaction influences the cellular death pathway further investigation is needed as well as more suitable model. It will be of great interest to study this interaction and SUMO role in brain/neurons both in pathological and physiological conditions.

Our findings are in contrast with some papers [32], [33] suggesting that SUMO-1 may play a protective role in models of oxidative stress. It is agreed that oxidative stress is complex and the data are rather confusing since high concentrations of H_2_O_2_ lead to increased SUMO conjugation while low H_2_O_2_ concentrations (1 mM and below) lead to almost complete loss of SUMO-1, -2 and -3 conjugates within the first 30 min [34]. In our model SUMO-1 conjugates remained stable, but the conditions and cells tested differ from the ones reported in the literature, thus it is very difficult to compare results.

In our model of oxidative stress inhibition of SUMOylation, with over-expression of the SENP1-de-SUMOylation enzyme, led to inhibition of JNK and to partial protection against H_2_O_2_-induced death. On the contrary, over-expression of SUMO-1 increased somewhat JNK activation and deteriorated cell death. This crosstalk between JNK and SUMO-1 pathways brought us to search a physical interaction connecting P-JNK to SUMO-1. Immunoprecipitation experiments convincingly proved that P-JNK interacts with SUMO-1 in both control (unstimulated) and H_2_O_2_-stimulated SH-SY5Y cells that overexpressed SUMO-1. Such interaction seems to increase in the presence of oxidative stress. These data were confirmed by immunostaining, where we could show that P-JNK colocalises with SUMO-1 in the nuclear bodies only in cells that are exposed to H_2_O_2_ stimulus. Altogether, this study is the first to present evidence for a direct interaction between JNK and SUMO in cellular death processes. The implication of this interaction requires further investigation. Although more studies are required it is tempting to speculate that such an interaction occurs also in neuronal cells and that deSUMOylation can be protective in neuronal loss occurring in several neurodegenerative diseases. Such studies are currently underway.

We here proved that SUMO interacts with the active form of JNK. For this reason and for the fact that the cell specific JNK inhibitor peptide totally prevented H_2_O_2_-induced cell death we suggest that SUMO-1 co-partecipates in the stress activated process by incrementing JNK activity.

This is the first report proposing that SUMO may not play a protective role but on the contrary can be implicated in death pathways.

## Materials and Methods

### Cell culture

SH-SY5Y [35] cells were maintained in complete medium with the following composition: DMEM (Invitrogen - GIBCO) 4,5 g/L glucose, 10% fetal bovine serum (LONZA SALES AG), 1% L-glutamine (Invitrogen - GIBCO) 200 mM, 1% antibiotics penicillin/streptomycin (Invitrogen - GIBCO), 1% MEM non-essential amino acids solution 100× (SIGMA).

Cells were seeded into 6-well plates at density of 4×10^5^ or 12-well plates at density of 2×10^5^ cells, one day before transfection.

Cells were incubated at 37°C under 5% CO_2_.

### Plasmids

pEYFP-SUMO-1 DGG was made by cloning human SUMO-1 into pEYFP-C1(Clontech) between KpnI and BamHI, pEGFP-SENP1 was produced cloning residue 351–644 (entire catalytic domain) of a human SENP1 into pEGFP-C1 (Clontech) using XhoI and EcoRI, FLAG-UBC9, pEYFP-SUMO-1DGG. All constructs were kind gifts by Jeremy HENLEY's lab (University of Bristol, Bristol, UK).

### Transfection and hydrogen peroxide stimulation

SH-SY5Y cells were transfected in OPTI-MEM+glutaMAX medium (Invitrogen – GIBCO) using Lipofectamine 2000 (Invitrogen) according to the manufacturer's procedure.

0.5 µg (for 6 wells) or 0.2 µg (for 12 wells) of each DNA was transfected per well. Empty plasmid was used where required to maintain equal total DNA quantity. 3 hours later transfection OPTI-MEM+glutaMAX medium was replaced by complete medium.

One day later, cells were treated with hydrogen peroxide (SIGMA) at different molarities for 3 h or O/N. SH-SY5Y cells were washed twice with PBS 1× (Invitrogen - GIBCO) and scraped on ice, span down and lysed in 100 µL Lysis Buffer solution (LB) made up of 1% Triton X-100 (Fluka), complete protease inhibitor cocktail tablets (Roche), phosphatase inhibitor cocktail tablets (Roche) and the following components (mM): TRIS acetate, 20; sucrose, 0.27; EDTA, 1; EGTA, 1; Na Orthovanadate, 1; NaF, 50; Na Pyrophosphate, 5; Na β-glycerophosphate, 10; DTT, 1.

### SDS-PAGE and Western blot

Samples containing 15–20 µg of protein were resolved by 8% denaturing SDS-PAGE. Proteins were transferred to PVDF membrane (BIO-RAD) and nonspecific binding sites were blocked with Tris-buffered saline–Tween (t-TBS; Tris, 0.02 M; NaCl, 0.150 M; and Tween 20, 0.1%) containing 5% non-fat dried milk.

The PVDF membranes were then probed with primary antibodies over night at 4°C. The primary antibodies and dilutions used were: Rabbit anti-SUMO-1 1∶1000 (Cell Signaling); Rabbit anti-P-JNKs 1∶1000 (Cell Signaling); Rabbit anti-JNKs 1∶1000 (Cell Signaling); Rabbit anti-P-c-JUN 1∶1000 (Cell Signaling); Rabbit anti-c-JUN 1∶1000 (Cell Signaling); Rabbit anti-GFP 1∶1000 (Abcam) and Mouse anti-actin 1∶20000 (Chemicon).

After washes membranes were incubated for 1 h at room temperature with appropriate secondary antibody: anti-Mouse, 1∶ 4000 (Santa Cruz Biotechnology) or anti-Rabbit, 1∶4000 (Santa Cruz Biotechnology) and anti-Rabbit Light Chain Specific (Jackson ImmunoResearch) for IP experiments. After washes immunoreactive bands were detected by enhanced chemiluminescence (ECL). In all experiments where an evaluation of phosphorylated proteins was required, antibodies were applied on the same membranes after stripping procedure (stripping buffer from Pierce). Actin was used as a loading control. The immunoreactive bands were visualized by exposure to Amersham Hyperfilm ECL (Ge Healthcare). Western blots were quantified by densitometric analysis using ImageJ software.

### MTT assay

MTT (Thiazolyl Blue Tetrazolium Bromide-SIGMA) colorimetric assay was employed to test cell viability in oxidative stress conditions. A 10× stock solution was prepared by dissolving 4 mg/mL of MTT powder in PBS 1× (Invitrogen – GIBCO).

200 µL MTT solution per well were added on cells, in 6-well plates, incubated at 37°C for 4 h. Medium was removed and cells were treated with a solution prepared by diluting 1∶25 HCl 1N with isopropanol (100%). Supernatants containing solubilized MTT crystals were analyzed at 540 nm to the spectrophotometer.

### Immunocytochemistry

Cells were plated in 12-well plates at density of 2×10^5^ cells above fixed by gelatine 0,1% (SIGMA) coverslips.

24 h after seeding medium was removed and cells were treated with H_2_O_2_ as required. Cells were then fixed in 4% paraformaldehyde-PBS for 15 minutes on ice, then permeabilized in PBS-Triton X-100 (Fluka) 0,3% for 15 minutes at room temperature. After 1 h blocking with serum 1% in PBS-Triton X-100 0,3%, primary antibodies were applied over night at 4°C at following concentrations: Mouse anti-P-JNKs 1∶100 (Santa Cruz Biotechnology), Rabbit anti-SUMO-1 1∶100 (Cell Signaling) all in 1% in NGS, Triton X-100 0,3% in PBS 1×. Secondary antibodies were then applied the day after for 2 h as required: anti-rabbit Alexa-488 (Green), anti-mouse Alexa-546 (Red).

Nuclear staining was obtained applying Hoechst solution (Roche) 1∶5000 for 5 min at room temperature. Cells were washed with 1× PBS after every step. Coverslips were mounted in Fluorsave mounting medium (Calbiochem, 345789). Staining was acquired with a Olympus microscope equipped with a Olympus Confocal scan unit (microscope BX61 and Confocal system FV500) managed by AnalySIS Fluoview software with 3 lasers line, UV-diode laser (405 nm), Ar–Kr (488 nm), He–Ne green (546 nm), respectively used to detect Hoechst staining and secondary antibody conjugated to Alexa 488 and Alexa 546. Double staining was revealed with a scanning sequential mode to eliminate possible bleed-through effect.

### Immunoprecipitation

Cells were lysed in 500 µL (around 300 µg of proteins) of Lysis Buffer (LB) and a preclearing step was performed adding for each sample 15 µL of washed (TBS buffer: 50 mM Tris, 150 mM NaCl pH 7.5) magnetic protein G sepharose beads (GE Healthcare) for 1 h at 4°C on wheel. Rabbit anti-P-JNK antibody (Cell Signaling; 1∶200) was then added to supernatants for 30 min at 4°C on wheel. 15 µL of washed beads where again added to each sample for O/N incubation. Beads were washed three times with TBS buffer and then 100 µL SDS-sample buffer was added. 20 µL of each sample was used for western blotting.

### Statistics

Statistical analysis was performed with GraphPad PRISM 5. Values shown represent the mean ± SEM of at least five separate experiments. One-way or Two-ways ANOVA followed by Tukey's test was carried out for intergroup comparisons were required.
